# Cholestasis and Seizure Due to Lead Toxicity: A Case Report

**DOI:** 10.5812/hepatmon.12427

**Published:** 2013-11-19

**Authors:** Ali Mokhtarifar, Hooman Mozaffari, Reza Afshari, Ladan Goshayeshi, Kambiz Akavan Rezayat, Kamran Ghaffarzadegan, Mohammadreza Sheikhian, Farnood Rajabzadeh

**Affiliations:** 1 Gastroenterology and Hepatology Department, Mashhad University of Medical Sciences, Mashhad, IR Iran; 2 Addiction Research Center, Mashhad University of Medical Sciences, Mashhad, IR Iran; 3 Radiology Department, Mashhad Branch, Islamic Azad University, Mashhad, IR Iran

**Keywords:** Lead, Cholestasis, Opium

## Abstract

**Introduction:**

Lead poisoning is a major public health risk which may involve major organs. Recently, there have been reports of opioid adulteration with lead in Iran. The following case report is the first of its kind in that intrahepatic cholestasis due to lead toxicity has been described.

**Case Presentation:**

A 65-year-old man presented to the emergency department with abdominal pain, abnormal liver function tests (cholestatic pattern), and normocytic anemia. He had been an opium user for 20 years. Clinical and preclinical findings including the bluish discoloration of periodontal tissues, or Burton’s sign, and generalized ileus on abdominal x-ray led us to the possibility of lead poisoning. Lead levels were higher than normal (150 μg/dL). Magnetic resonance cholangiopancreatography (MRCP) and abdominal ultrasound were performed to rule out extra hepatic causes of cholestasis. To evaluate the possibility of lead-induced hepatotoxicity, a liver biopsy was performed. Histological features of lead-induced hepatotoxity have rarely been described in humans. In this patient, focal canalicular cholestasis and mild portal inflammation were confirmed. Thus, treatment with ethylenediaminetetraacetic acid (EDTA) and British anti-lewisite (BAL) were initiated and continued for five days. The patient’s liver function tests returned to their normal values, clinical findings including nausea, vomiting, and abdominal pain subsided, and the patient was discharged from the hospital in good condition.

**Conclusions:**

Lead toxicity should always be taken into account in cases of intrahepatic cholestasis with an unknown etiology, especially in a setting where opium abuse is common.

## 1. Introduction

Lead is available in the environment widely. Lead poisoning has been recognized as a major public health risk, particularly in developing countries (1). Opioid abuse is common in Iran (2). Opioid adulteration including lead, thallium, and steroids are commonly reported (3-5). It has been shown that adulteration of lead opioids could result in severe lead toxicities (5, 6).

Lead affects major organ systems in the body including hematopoietic, gastrointestinal, respiratory, renal, nervous and cardiovascular mainly through increased oxidative stress, ionic mechanisms and apoptosis (1, 7-10).

Lead exposure occurs mainly through the respiratory and gastrointestinal systems, and it is stored in soft tissues as well as bones. Autopsy studies have suggested that 33% of the absorbed lead is stored in the liver (10). Ingestion of lead is one of the primary causes of its hepatotoxic effects (1, 10).

Nowadays, there are very few case reports which have described lead induced hepatotoxity in humans. This is the first case in which intrahepatic cholestasis due to lead toxicity is described (11, 12). Although, acute abdominal pain of unclear origin, caused by lead poisoning in patients with opium addiction, has been reported (13, 14).

## 2. Case Presentation 

A 55-year-old man presented to the emergency department at Imam Reza Hospital in Mashhad with abdominal pain, icterus and high serum alkaline phosphatase levels. He had been a raw opium addict for over 20 years. He had undergone cholecystectomy three weeks prior to admission for gallbladder stones, but his pain did not resolve after the surgery. He was admitted on suspicion of remnant common bile duct (CBD) stones after cholecystectomy.

On admission, he complained of abdominal pain of one month’s duration. The pain was constant and severe and usually lasted 1 to 2 hours. The pain was located in the right upper quadrant (RUQ) and periumbelical regions. He also reported postprandial nausea and vomiting. He had no defecation for eight days but did report gas passage. He was also chronically constipated for a period of one year with limited response to common medications. There was no history of pruritus or change in urinary or stool color.

**Figure 1.**
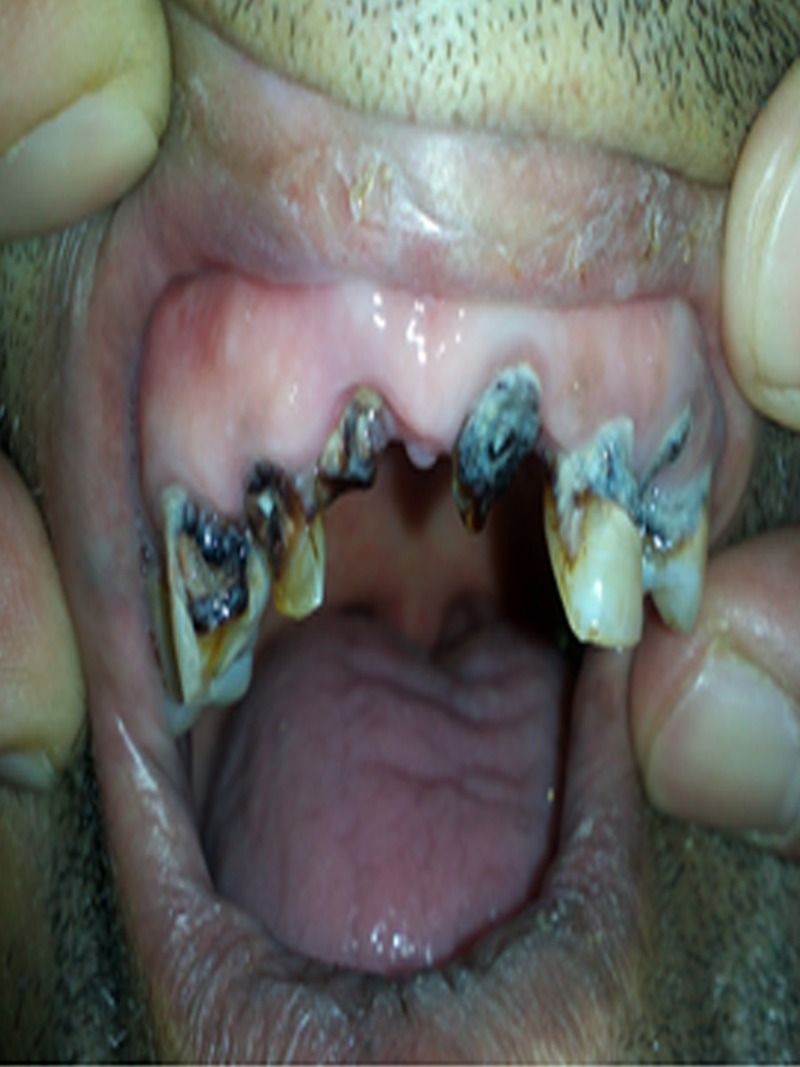
Bluish discoloration of periodontal tissues (Burton sign)

The patient’s physical examination revealed stable hemodynamics, with a bluish discoloration of periodontal tissue (Figure 1) and mildly icteric sclera. The chest examination was normal. The abdomen was distended with diminished bowel sounds and mild tenderness to deep palpation of the periumbelical and RUQ regions without rebound or guarding. Fecal material was detected on the digital rectal examination. The neurological examination was unremarkable.

Laboratory findings included a normocytic anemia (hemoglobin: 8.2 g/dL; mean corpuscular volume [MCV]: 82); a normal leukocyte count and differentiation; aspartate transaminase (AST): 100 IU/L; alanine transaminase (ALT): 76 IU/L; serum ALP: 3100 IU/L; gamma glutamyl transpeptidase (GGT): 1057 IU/L (normal range up to 49 IU/L); total bilirubin: 3.5 mg/dL; direct bilirubin: 1.7 mg/dL; erythrocyte sedimentation rate (ESR): 31 mm/hr; and creatinin (Cr): 0.8 mg/dL. The urine analysis and serum sodium, potassium, magnesium and calcium levels were within the normal range.

The initial differential diagnoses based on the patient’s symptoms and signs and laboratory findings included remnant stones in the CBD, post-surgical complications, a dysfunctional sphincter of Oddi associated with opium addiction, and generalized ileus due to recent surgery.

**Figure 2.**
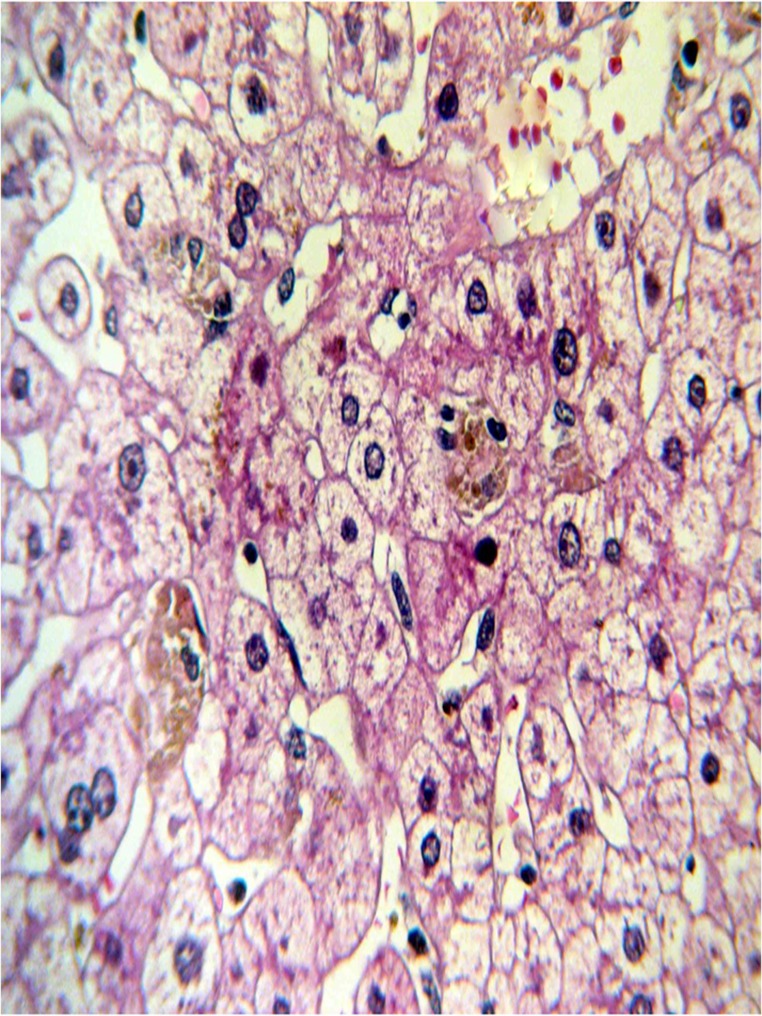
Liver biopsy showing mild canalicular cholestasis and feathery degeneration

**Figure 3.**
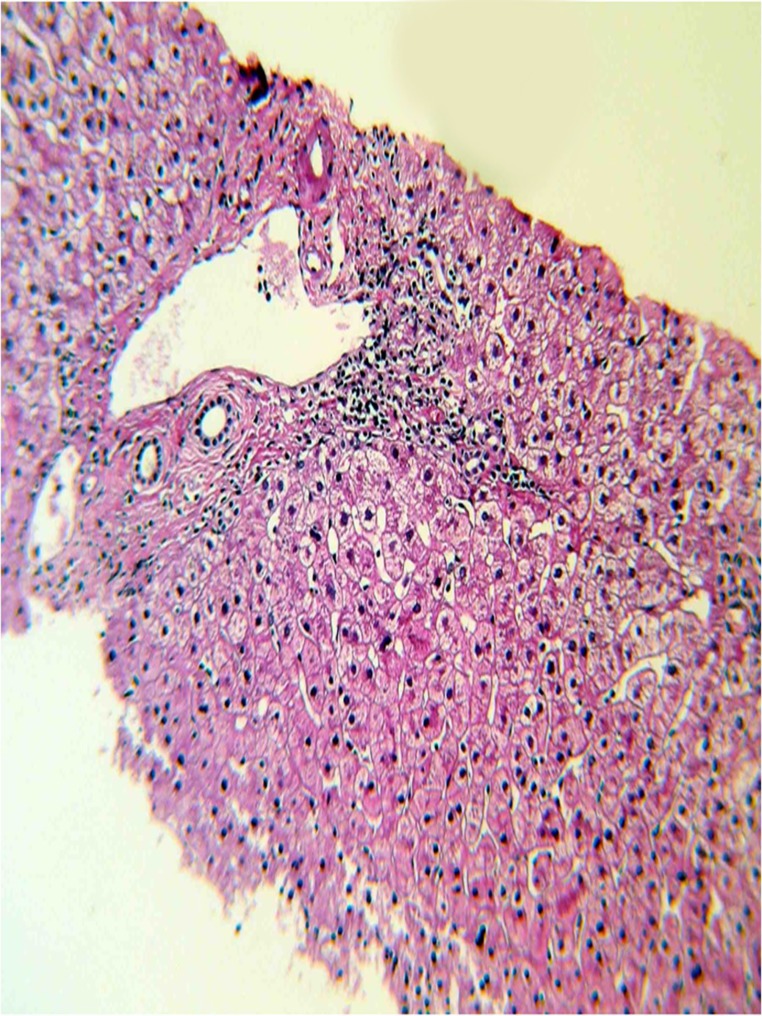
Liver biopsy showing portal spaces with mild mononuclear portal inflammation

Plain abdominal x-ray showed generalized ileus with fecal impaction. Ultrasound study of the liver revealed mild fatty liver changes with normal biliary ducts. Therefore, extrahepatic causes of cholestasis were ruled out. There was no history of drug consumption in the recent months to justify related potential intrahepatic cholestasis. Viral serology was negative for hepatitis A, B or C. Antinuclear antibodies (ANA), anti-smooth muscle antibodies (ASMA), anti-mitochondrial antibodies (AMA), anti-liver kidney microsome 1 antibodies (LKM 1), perinuclear anti-neutrophil cytoplasmic antibodies (P-ANCA) were all within normal limits and the serum protein electrophoresis was unremarkable. Thus, other potential causes of intrahepatic cholestasis including sepsis, viral hepatitis, autoimmune diseases, primary biliary cirrhosis (PBC) and primary sclerosing cholangitis( PSC) were ruled out with proper laboratory and imaging (i.e. MRCP) modalities. Subsequently, liver biopsy was performed to investigate the remained unestablished cause of the patient’s intrahepatic cholestasis. The pathology report was indicative of a preserved architecture; mild lymphocytic mononuclear infiltration in the portal spaces; foci of canalicular cholestasis, mostly of zone 3; and areas of cells with glycogenated nuclei, hence nonspecific hepatitis (Figure 2, 3).

Four days into admission the patient had severe nausea and vomiting and one episode of generalized tonic colonic seizure. Neurologic investigations including brain computed tomography scan, brain magnetic resonance imaging, and magnetic resonance venography were normal. The patient did not consent to lumbar puncture.

Causes of anemia including iron deficiency and hemolytic anemia were excluded with serum iron and ferritin levels (253 µg/dL and 1139 ng/mL, respectivley), a TIBC of 457 µg/dL, and normal serum lactate dehydrogenase levels (322 IU/L).

Lead poisoning as the culprit for the patient’s anemia was suspected only later, when bluish discolorations of the periodontal tissues and abdominal cramps were considered in association with the patient’s history of opium addiction. The patient’s other clinical findings were consistent with lead toxicity. The diagnosis of lead toxicity was confirmed through serum lead level measurements – whole blood lead levels of 150 µg/dL. Investigation of the patient’s past history failed to reveal any exposure to lead but the use of opium; opioid adulteration with lead in the area has previously been reported (5, 6, 14-16).

According to the toxicology consultation, EDTA and BAL were administered for five days. The use of raw opium was discontinued and methadone was administered for the withdrawal symptoms.

After initial treatment, serum lead levels decreased to 41 µg/dL and by the last day of admission 24-hour urinary lead levels reached 2080 mg/dL, liver function tests had returned to normal values (AST: 46 IU/Dl; ALT: 48 IU/dL; ALP: 269 IU/dL; total bilirubin: 0.5 mg/dL; and direct bilirubin: 0.1mg/dL), and the hemoglobin (Hb) was 10.3 gm/dl. In addition, clinical findings including nausea, vomiting and abdominal pain had subsided without any recurrence of seizures. Ultimately, the patient was discharged from the hospital in good condition and was told to monthly visits for six months. The resulted laboratory findings are summarized in Table 1.

**Table 1.** Variables in the Patient With Chelation Therapy During Follow Up

	Day 1	Day 3	Day 6	Day 9	Day 12	Day 50
**AST, IU/dl** ^**a**^	136	138	111	58	59	35
**ALT, IU/dl ** ^**a**^	100	99	109	81	50	42
**ALP, IU/dl ** ^**a**^	2452	1592	1354	943	375	679
**Total Bilirubin , mg/dl** ^**a**^ **, mg/dl**	1.8	1.5	1.2	101	0.8	0.7
**Direct Bilirubin, mg/dl** ^**a**^ **), mg/dl**	0.7	0.5	0.4	0.4	0.3	0.2
**Serum lead level, microgram/dl**	1500				420	360

^a^Abbreviations: ALT, Alanine amino Transferase; ALP, alkhaline phosphatase; AST, Aspartate amino Transferase

## 3. Conclusions

The report is another piece of evidence showing the relationship between opium abuse and lead toxicity. While hepatotoxicity due to lead exposure has been reported (11, 12), this is the first description of lead-toxicity-induced cholestasis.

Lead poisoning should be considered in the differential diagnosis in patients presenting with Burton’s sign, abdominal pain and anemia, particularly in areas in which opioid use is common. This is similar to previous reports (5, 6, 11, 13). There was no known exposure to lead which is found in the patient’s history and the most likely source of lead toxicity seemed to be opium.

Lead-induced histopathological changes in the liver have been reported in animal studies in geese and dogs (17, 18). Hemosiderosis, bile plugs in dilated canaliculi, bile pigmentation in hepatocytes, and hepatic necrosis have been described in liver biopsies from such animals (17). Elevated serum levels of ALP, lactate dehydrogenase, and GGT have been reported in lead poisoning (10).

Lead induced hepatotoxicity is very rare in humans. There is a report which is published 30 years ago of four individuals who had committed suicide by self-injecting lead and taking opium pills prior to drowning. They had developed general malaise, vomiting and constipation. Serum lead, AST and ALP levels were elevated. Liver biopsies were indicative of nonspecific hepatitis. Recently, high lead levels were reported in the blood of a male opium addict with abdominal pain and abnormal liver function tests. The liver biopsy in this case revealed active hepatitis with extensive microvesicular steatosis, hemosiderosis, and cholestasis with lymphocytic cholangitis (11).

In the present study, intrahepatic cholestasis with high serum ALP and GGT levels was detected. In the liver biopsy mild lymphocytic mononuclear infiltration in the portal spaces, foci of canalicular cholestasis, mostly of zone 3, and areas of cells with glycogenated nuclei were reported. These findings were similar to previous reports of lead-toxicity-induced microscopic hepatic changes.

The patient had two episodes of tonic colonic seizures and lower limb paresthesia. Brain imaging was normal. Lead induced encephalopathy could be the most probable cause. Encephalopathy due to lead toxicity has been observed in patients after exposure to high concentrations of lead (10). Both central and peripheral nervous systems could be affected by lead. However, involvement of the peripheral nervous system is more common in adults while in children the central nervous system is affected more frequently (1, 10). Symptoms of lead-induced encephalopathy are dullness, irritability, poor attention span, headache, muscular tremor, loss of memory and hallucinations. More severe manifestations occur at very high serum lead levels and include convulsion and coma (10).

In the present case serum lead levels were 150 µg/dL. The patient reported dullness, poor attention and fatigue for a year. Nausea, vomiting, and paresthesia of the lower limbs as well as two episodes of convulsions occurred during admission.

Lead directly affects the hematopoietic system by preventing the synthesis of hemoglobin and reducing the life span of erythrocytes, thus leading to anemia (1, 10, 11).

Hemolysis results in high iron deposition in the reticulo-endoplasmic system and an increase in the serum values of ferritin and iron saturation together with a decrease in iron binding capacity (1, 11, 19). In this patient, a normocytic anemia and high ferritin levels were detected. The anemia resolved with treatment.

Opium adulteration with lead is commonplace in Iran (5, 6, 15, 16). Recently, there have been reports related to lead poisoning as a consequence of opium addiction (5, 6, 11, 13, 14, 16).

In conclusion, in cases with severe abdominal pain and ileus particularly with concomitant anemia, lead poisoning should be included in the differential diagnosis. This condition could rarely be accompanied by hepatitis, cholestasis, and neurologic signs. In patients with intrahepatic cholestasis with an unknown etiology, lead toxicity should be considered, especially in a setting where opium abuse is common.
